# Pattern and outcome of acute kidney injury among Sudanese adults admitted to a tertiary level hospital: a retrospective cohort study

**DOI:** 10.11604/pamj.2017.28.90.11054

**Published:** 2017-09-29

**Authors:** Marwa Osman, Mazin Shigidi, Haider Ahmed, Ihab Abdelrahman, Wieam Karrar, Elhussein Elhassan, Hussam Shwaib, Rayyan Ibrahim, Marwa Abdalla

**Affiliations:** 1 Department of Community Medicine, Faculty of Medicine University of Khartoum, Sudan; 2 Dr. Salma Centre for Kidney Diseases, Faculty of Medicine, University of Khartoum, Sudan; 3 Department of Medicine, Faculty of Medicine University of Khartoum, Sudan; 4 Ministry of Health, Khartoum, Sudan

**Keywords:** Acute kidney injury, etiology, outcome, adults, Sudan

## Abstract

**Introduction:**

Little is known about the pattern and outcome of Acute Kidney injury (AKI) in Sudan. This study aimed to determine the etiology and outcome of AKI among Sudanese adults.

**Methods:**

A retrospective cohort study was conducted in a tertiary level hospital, Soba University Hospital, Sudan. The medical records of all adults admitted to hospital from the 1^st^ of January to 31^st^ of December 2014 were reviewed. The diagnosis and severity of AKI was defined as per the Kidney Disease Improving Global Outcomes (KDIGO) recommendations.

**Results:**

The medical records of 6769 patients were reviewed. AKI was diagnosed in 384 patients (5.7%); being community acquired in 82.6% of cases. Sepsis, volume depletion, obstructive uropathy, heart failure, acute glomerulonephritis and severe malaria were the commonest causes of AKI diagnosed in 44%, 38.5%, 8.9%, 5.7%, 4.7% and 3.1% of patients, respectively. Following treatment complete renal recovery was seen in 35.7% of patients; whereas 31.2% of patients died. Predictors of increased risk of death were old age [OR 1.03, 95% CI (1.01-1.057); P=0.003], presence of chronic liver disease [OR 2.877, 95% CI (1.5-5.5); P=0.001], sepsis [OR 2.51, 95% CI (1.912-4.493);P=0.002] and the severity of AKI [OR 3.873, 95% CI(1.498-10.013);P=0.005].

**Conclusion:**

AKI was diagnosed in 5.7% of adults admitted to hospital. Most patients were having community acquired AKI. Old age, the presence of chronic liver disease, sepsis, and the severity of AKI as per KDIQO staging were significant predictors of mortality.

## Introduction

It had been estimated that around 1 in every 5 adults admitted to hospital develop Acute Kidney Injury (AKI) [1]. In hospital, AKI is known to be significantly associated with lengthy admissions, greater medical cost and increased patients’ morbidity and mortality [1–5]. Most of the data available in the literature regarding the incidence, etiology and outcomeof AKI were reported by the developed countries [6]. The pattern of AKI in Africa is thought to be different as prevalent illnesses such as human immunodeficiency virus infection, diarrheal diseases, malaria, nephrotoxins and obstetric complications are leading causes of AKI among hospitalized patients. Furthermore, the delayed presentation of AKI patients to health care facilities, lack of resources, late recognition of the disease and absence of reliable statistical data regarding the incidence of AKI all add to the magnitude of the problem in developing countries [6–8]. In Sudan, little data had been published regarding the epidemiology of AKI and its prognosis [9, 10]. This study aimed to determine the incidence, etiology, risk factors, prognostic parameters and outcome of AKI among Sudanese adults admitted to a tertiary level hospital in central Sudan.

## Methods

A retrospective, hospital based cohort study was done in Soba University Hospital (SUH), Khartoum, Sudan. SUH is a 400-bed capacity specialized referral hospital, located at the southern sector of the capital city Khartoum State. The hospital receives referred cases mostly from the capital city and central Sudan; an area including a population with great ethnic diversity from the different parts of the country [11].

Besides being a tertiary referral hospital that provide medical, surgical and obstetrical services on elective and emergency basis; SUH was specifically selected for this study as it includes an active internal medicine department with a highly specialized nephrology division that provide renal services as per consultation. The hospital is known to have an accessible, good quality hospital medical records system designed for research purposes.

The medical records of all adult patients admitted to SUH during the period from January 1^st^, 2014 to December 31^st^, 2014 were extracted and reviewed for the presence or diagnosis of AKI. The diagnosis of AKI and its severity were set using the Kidney Disease Improving Global Outcomes (KDIGO) definition and staging criteria [12, 13]. The medical records of those less than 18 years of age, readmissions of the year 2013, those diagnosed as having AKI outside the study period and patients with end stage renal disease were excluded from the study. Records with inconclusive medical data regarding the diagnosis of AKI were also excluded.

Data collection was carried out by 6 resident doctors using a specially designed and pre-tested questionnaire. Targeted information from the hospital paper-based medical records included patients’ demographic data, risk factors for developing AKI, etiology and severity of AKI, treatment given, pattern of renal recovery and the overall patients´ outcome. The severity of AKI was graded from 1 to 3 as per the KDIGO recommendations [12, 13]. AKI was stated as fully recovered once the serum creatinine had returned to levels within the normal laboratory reference values (0.4 - 1.4mg/dl) or its prior baseline levels. Patients were labeled as having partial renal recovery if the serum creatinine dropped down-to-short of the normal or baseline levels. No renal recovery was diagnosed if the serum creatinine did not decrease or continued to rise till the time of discharge or death.

Data analysis was done using Statistical Package for Social Science (SPSS) version 20. Descriptive analysis of continuous data was applied using means and medians with standard deviations (SD) and interquartile range (IQR), respectively. Chi square test and logistic regression analysis were applied for categorical data with the levels of significance being setat 0.05. The study was approved by the ethical committee of the Department of Community Medicine, University of Khartoum. Ethical clearance was also obtained from the Soba University Hospital, University of Khartoum, Sudan.

## Results

The medical records of 6769 adults admitted to SUH, during the year 2014, were reviewed. Among these the diagnosis of AKI was evident in 384 files; thus making an annual incidence of AKI of 5.7% of all hospital admissions. On average a total of 32 ± 5 patients with AKI were admitted monthly to SUH. No seasonal variations were seen in the number of AKI admissions along the year, with a P value of 0.6. Most patients, 82.6%, were having community acquired AKI. Hospital acquired AKI was diagnosed in 16.9% of patients. It was difficult to determine whether it was hospital or community acquired AKI in the records of 2 patients, 0.5%.

AKI was predominantly diagnosed in the general medical ward, 83.8%; with lesser number of AKI patients being seen in the surgical and obstetrical wards accounting for 8.6% and 1.6% of cases, respectively. The median age of AKI patients was 57 years (IQR 41 and 70). Most patients, 54.9%, were referred from rural areas. More than 65% of patients with AKI were males; predominance of the male sex was statistically significant, with a P value of 0.001 (Table 1).

**Table 1:** incidence and type of acute kidney injury among Sudanese adults admitted to a tertiary level hospital

Features	Frequency (%)
Incidence of ^+^AKI	384 (5.7%) / 6769 (100%)
**Type of ^+^AKI diagnosed**	
Community acquired	317 (82.6%)
Hospital acquired	65 (16.9%)
Uncertain classification	2 (0.5%)
**Admitting hospital department**	
Medical ward	322 (83.8%)
Surgical ward	33 (8.6%)
Intensive Care Unit	23 (6%)
Obstetrical ward	6 (1.6%)
Median age of the study population	57 years (IQR 41 and 70)
Male / Female ratio	252 (65.6%) / 132 (34.4%)
Urban / rural residency	126 (32.8%) / 211(54.9%)

+ Acute Kidney Injury

**Figure 1:**
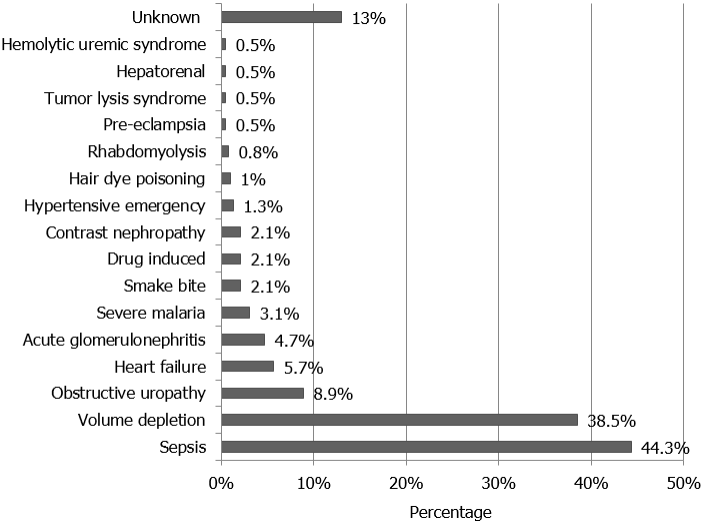
Causes of acute kidney injury among Sudanese adults admitted to a tertiary level hospital

**Figure 2:**
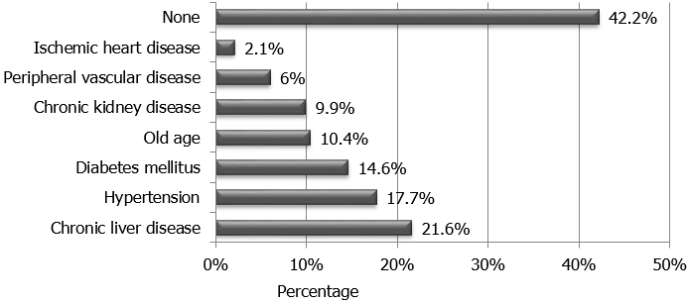
Risk factors for developing acute kidney injury among Sudanese adults admitted to a tertiary level hospital

Sepsis, volume depletion, obstructive uropathy, heart failure, acute glomerulonephritis and severe malaria were the commonest causes of AKI seen in 44.3%, 38.5%, 8.9%, 5.7%, 4.7% and 3.1%, of cases, respectively (Figure 1). Risk factors for developing AKI included chronic liver disease, hypertension, diabetes mellitus, old age and presence of a background chronic kidney disease seen in 21.6%, 17.7%, 14.6%, 10.4%, and 9.9% of the patients studied, respectively (Figure 2).

As per the KDIGO staging 24.2% of patients were graded as having AKI stage 1, 27.9% AKI stage 2 and 47.9% AKI stage 3. Nineteen percent of AKI patients required dialysis therapy. One patient had peritoneal dialysis, whereas all other patients were treated via intermittent hemodialysis with a median number of 3 hemodialysis sessions (IQR 2 and 4) being required. The median duration of hospital stay for AKI patients was 9 days (IQR 5 and 14). Full renal recovery was seen in 35.7% of patients. Partial or no renal recovery during hospital admission was seen in 45% of patients; all being referred for outpatient follow-up at nephrology clinic. The outcome of AKI could not be traced in the medical records of 18.8% of patients, thus labeled as having unknown outcome. Regarding the overall patients’ outcome, 59.6% of patients survived their acute illness; whereas 31.2% died during admission. Patients’ outcome was not specified, whether survived or died, in the records of 35 patients, 9.1% (Table 2).

**Table 2:** Severity and outcome of acute kidney injury among Sudanese adults in a tertiary level hospital

Features	Frequency (%)
**Severity of ^+^AKI as per ^†^KDIGO Staging**	
AKI stage I	93 (24.2%)
AKI stage 2	107 (27.9%)
AKI stage 3	184 (47.9%)
Total number of patients required dialysis	73 (19%)
**Outcome of AKI**	
Fully recovered	137 (35.7%)
Showed partial recovery	80 (20.8%)
Showed no renal recovery	93 (24.2%)
Diagnosed as developed ^‡^CKD	2 (0.5%)
Unknown	72 (18.8%)
Median duration of hospital stay	9 days (IQR 5 and 14)
**Overall patients’ outcome**	
Recovered	229 (59.6%)
Died in hospital	120 (31.2%)
Unknown	35 (9.1%)

+ Acute Kidney Injury;

† Kidney Disease Improving Global Outcomes;

‡ Chronic Kidney Disease

In order to determine the predictors of requirement of dialysis therapy among AKI patients, binary logistic regression analysis was implemented on our data using patients’ demographic features, risk factors for developing AKI and the etiology of AKI as independent variables. Requirement of dialysis among our patients significantly correlated with the increase in patients’ age [OR, 1.02; 95% CI (1.006-1.048); P = 0.012], presence of background chronic kidney disease [OR, 6.34; 95% CI (2.765-15.49); P = 0.0001], absence of hypovolemia [OR, 0.357; 95% CI (0.169-0.754); P = 0.007], and the occurrence of AKI due to obstructive uropathy [OR, 3.423; 95% CI (1.293-9.05); P = 0.013], snake bite [OR, 6.058; 95% CI (1.201-30.565); P = 0.029] and hair dye poisoning [OR, 11.205; 95% CI (1.038-120.952); P = 0.047].

On the other hand, binary logistic regression was implemented further to determine the independent predictors of mortality among AKI patients; accordingly patients’ mortality significantly correlated with the increase in patients’ age [OR, 1.03; 95% CI (1.01-1.057); P = 0.003], presence of chronic liver disease [OR, 2.877;95% CI (1.5-5.5); P = 0.001], presence of sepsis [OR, 2.51;95% CI(1.912 - 4.493); P = 0.002] and the severity of AKI as per the KDIGO staging [OR 3.873;95% CI (1.498- 10.013); P = 0.005] (Table 3).

**Table 3:** Predictors of requirement of dialysis and increased mortality among AKI patients

Predictors of outcome	Odd ratio	CI^†^ low	CI^†^ high
Predictors of requirement of dialysis			
Old age	1.02	1.006	1.048
Background kidney disease	6.34	2.76	15.49
Absence of hypovolemia	0.36	0.17	0.75
AKI due to obstructive uropathy	0.36	1.29	9.05
AKI due to snake bite	6.06	1.2	30.57
AKI due hair dye poisoning	11.21	1.04	120.95
Predictors of increased mortality			
Old age	1.03	1.01	1.06
Presence of chronic liver disease	2.88	1.5	5.5
AKI due to sepsis	2.51	1.91	4.49
Severity of AKI as per KDIQO staging	3.87	1.5	10.01

† Confidence Interval

## Discussion

Most of the published data estimating the incidence of AKI were from high income countries and including patients admitted to critical care units; settings where the ability to trace and detect suspected cases remains high. In a systematic review by Susantitaphong *et al*. the world incidence of AKI among hospitalized adults was estimated to be 21.6%, and the condition was found to be associated with a 23.9% increase in mortality [1]. The data available in the literature regarding the incidence and outcome of AKI varies widely and is greatly influenced by the populations studied, definitions of AKI applied, quality of health care provided, and awareness of health care providers regarding the occurrence of AKI [1, 14].

In Africa, AKI was reported to account for 3% of admissions to hospital [8]. It is believed that the true incidence is much higher than that estimated, mostly due to the under-representation of reports from African countries in published reviews [1]. Sepsis and volume depletion were the commonest cause of AKI reported in Africa [8, 15]; whereas hypertension, diabetes mellitus, cardiovascular diseases, chronic kidney disease and old age were the commonest risk factors for the development of the disease [16, 17]. Those who are at high risk for developing AKI are expected to benefit from early identification and monitoring of their renal function once admitted to hospital. The presence of risk factors on risk assessment should always alert the attending physician regarding the importance of monitoring the kidney function once patients are admitted and throughout their hospital stay; such an intervention is expected to allow for early detection of AKI, decrease its morbidity and mortality [18]. This attitude had always been prevalent in the medical rather than the surgical or obstetrical wards allowing for the relatively higher incidence of AKI detected in the general medical wards [18, 19].

In SUH, AKI was reported in 5.7% of admissions with most patients being referred from rural areas and with community acquired AKI. These findings are consistent with previous reports which described AKI in developing countries as being community acquired and mostly referred from rural areas, with lesser patients being diagnosed as having hospital acquired AKI following surgical or obstetrical complications [3, 8, 19]. The median age of AKI patients was 57 years, a younger age group compared to that reported from developed countries [19–21]. In a series of epidemiological studies from Europe and the United States, the median age of adult patients admitted to hospital with AKI were reported as 77 and 72 years, thus labelling AKI as the disease of the elderly [20, 21].

Among the study population chronic liver disease, hypertension, diabetes mellitus, old age and chronic kidney disease were the dominant risk factor for developing AKI. In central Sudan, chronic liver disease due to schistosomal periportal fibrosis remains a major health concern, being schistosomiasis highly endemic in the region with an overall prevalence of Schistosoma *mansoni* reaching 68.5% in some districts. Patients with schistosomal periportal fibrosis are frequently admitted to hospital with decompensated liver disease, hematemesis and subsequently AKI [22, 23].

Patients’ mortality following AKI was reported to approach 28.2%, 28.1% and 23.9% in Qatar, the United Kingdom and China; respectively [19, 24, 25]. Again, full renal recovery upon discharge tends to reach 54% among patients with community acquired AKI and 45% among those admitted with hospital acquired AKI; with a median length of hospital stay of 7 and 15 days for both groups, respectively [19]. In SUH, complete renal recovery upon discharge was evident in 35.7% of AKI patients; whereas 31.2% died during their course of illness. Among those who died the presence of chronic liver disease, sepsis, old age and the severity of AKI as per the KDIGO staging were significant predictors of increased mortality. Most AKI patients studied, 47.9%, were having AKI stage 3 as per the KDIGO grading of severity; forty percent of these patients required dialysis therapy. It is this group of patients who had the longest hospital stay, highest cost of treatment and increased patients´ morbidity [26].

Serum creatinine remains the classical biomarker for diagnosing AKI. In SUH serum creatinine level is requested for every patient upon admission except the pregnant females admitted for delivery with no risk factors. Throughout the study, the diagnosis of AKI and its outcome were determined by the serum creatinine levels obtained from the patients’ medical records, these were not only related to the prior request of the test upon admission but also the presence of the results in the medical records. Limitations of this study include its retrospective nature. The medical record system is paper-based and the review process had to be done manually. The presence of incomplete records, poor handwriting and variability of the terms used in the patients’ files were all factors limiting the review process. In this study patients’ follow-up was restricted to the admission period and does not predict the long-term patient survival and renal outcome. Multicenter, prospective long-term studies are recommended to determine the etiology and prognosis of AKI among Sudanese adults.

## Conclusion

Among Sudanese adults, 5.7% of patients admitted to a tertiary level hospital were having AKI. The commonest causes of AKI were sepsis, volume depletion and obstructive uropathy seen in 44.3%, 38.5% and 8.9% of patients, respectively. Major risk factors for developing AKI include chronic liver disease, hypertension and diabetes mellitus. Dialysis replacement therapy was required in 19% of AKI patients admitted to hospital. Death was reported in 31.2% of AKI patients. Predictors of increased mortality were old age, the presence of chronic liver disease, sepsis, and severity of AKI as per the KDIGO staging criteria.

### What is known about this topic

- Most of the data regarding AKI were from developed countries;
- AKI is associated with increased morbidity, mortality and prolonged hospital admissions;
- In Africa AKI is under-reported with most cases being passed unnoticed.

### What this study adds

- Among Sudanese adults 5.7% of hospital admissions were diagnosed as having AKI, which is predominantly community acquired;
- Sepsis and volume depletion were the commonest causes of AKI among Sudanese adults admitted to hospital;
- Most patients admitted with AKI were found to have the severest form of the disease, KDIQO stage 3.

## Competing interests

The authors declare no competing interests.
